# *Bacillus subtilis,* the model Gram‐positive bacterium: 20 years of annotation refinement

**DOI:** 10.1111/1751-7915.13043

**Published:** 2017-12-26

**Authors:** Rainer Borriss, Antoine Danchin, Colin R. Harwood, Claudine Médigue, Eduardo P.C. Rocha, Agnieszka Sekowska, David Vallenet

**Affiliations:** ^1^ Department of Phytomedicine Humboldt‐Universität zu Berlin Lentzeallee 55‐57 14195 Berlin Germany; ^2^ Hôpital de la Pitié‐Salpêtrière Institute of Cardiometabolism and Nutrition 47 Boulevard de l'Hôpital 75013 Paris France; ^3^ School of Biomedical Sciences Li Kashing Faculty of Medicine University of Hong Kong 21 Sassoon Road Pok Fu Lam SAR Hong Kong China; ^4^ The Centre for Bacterial Cell Biology Newcastle University Baddiley‐Clark Building Richardson Road Newcastle upon Tyne NE2 4AX UK; ^5^ CEA DRF Genoscope LABGeM CNRS, UMR8030 Génomique Métabolique Université d'Evry Val d'Essonne Université Paris‐Saclay F‐91057 Evry France; ^6^ Microbial Evolutionary Genomics Unit Institut Pasteur 28 rue du Docteur Roux 75724 Paris Cedex 15 France

## Abstract

Genome annotation is, nowadays, performed via automatic pipelines that cannot discriminate between right and wrong annotations. Given their importance in increasing the accuracy of the genome annotations of other organisms, it is critical that the annotations of model organisms reflect the current annotation gold standard. The genome of *Bacillus subtilis* strain 168 was sequenced twenty years ago. Using a combination of inductive, deductive and abductive reasoning, we present a unique, manually curated annotation, essentially based on experimental data. This reveals how this bacterium lives in a plant niche, while carrying a paleome operating system common to Firmicutes and Tenericutes. Dozens of new genomic objects and an extensive literature survey have been included for the sequence available at the INSDC (AccNum AL009126.3). We also propose an extension to Demerec's nomenclature rules that will help investigators connect to this type of curated annotation via the use of common gene names.

## Introduction

With the advent of Next Generation Sequencing (NGS) techniques, sequencing genomes has become routine. While this is of tremendous interest by providing a profusion of sequence data, contributing accurate knowledge coupled to the sequences has become a nightmare (Zallot *et al*., 2016). The main reason for this dire state of affairs is that automated *in silico* pipelines draw ‘knowledge’ by inference, relying primarily on protein sequence similarity analysis, with the function tag extracted from databases that basically lack experimental information. Worse, this approach most often uses the majority rule (a function is deemed correct if it is found in the majority of annotations). Several correction systems have been devised to improve this highly inadequate approach, but the fact is that in the absence of a process allowing experimental validation (direct or indirect) of annotations, errors continue to percolate through the system (Gilks *et al*., 2005). It is therefore of the utmost importance that, for at least some reference genomes, manual curation of sequence data be still maintained on a routine basis (Chang *et al*., 2016). Unfortunately, there is little or no reward for this type of work despite the fact that individual scientist still develops much of their research and make key discoveries based on knowledge rooted in sequence annotations. Briefly, investigators demand access to knowledge, but they are extremely reluctant to pay for that access in any way. The consequence is that, at the present time, the number of cleanly annotated genomes is vanishingly small (of note are the EcoGene resource for *Escherichia coli* (Zhou and Rudd, 2013), a recent update for the genome of *Pseudomonas putida* KT2440 (Belda *et al*., 2016) and ongoing work on *Mycobacterium tuberculosis* (Lew *et al*., 2013)). *Bacillus subtilis*, strain 168 remains a case in point, and here we present an updated annotation, based on experimental evidence collected for this organism but also from other organisms, that we describe here with the aim of summarizing knowledge about this bacterium as a possible chassis for Synthetic Biology studies.

The genome sequence of *B. subtilis* 168 was published in 1997 by a consortium mainly formed by European and Japanese laboratories (Harwood and Wipat, 1996; Kunst *et al*., 1997). At the time, sequencing was very hard work because it primarily rested on cloning fragments of DNA into an *E. coli* recipient host before sequencing, under conditions where at least 15% of the sequences failed to be cloned. The reason of this unwelcome difficulty was that transcription and translation signals in *B. subtilis* are unexpectedly efficient in *E. coli*, resulting in toxic levels of gene expression, particularly of membrane proteins (Frangeul *et al*., 1999). The situation improved when, late in the project, long‐range PCR became routine. The sequencing of genomes of similar composition remained fairly intractable. This resulted in the genome of *B. subtilis* being the only Firmicute genome sequence for almost five years, until those of the much smaller genomes of *Staphylococcus aureus* (Kuroda *et al*., 2001) and *Streptococcus pneumoniae* (Hoskins *et al*., 2001; Tettelin *et al*., 2001) were published, followed by that of *Bacillus anthracis* strains of size equivalent to that of *B. subtilis* (Read *et al*., 2002).

Being one of the two very first bacterial genomes longer than 4 Mb to be sequenced implied that an appreciable level of errors must have crept in. This was expected because the sequence was obtained in different laboratories, where a variety of experimental protocols was used. It is also likely that mutations occurred even during the cultivation steps that are a prerequisite to sequencing. The genome was therefore entirely re‐sequenced using NGS methods ten years later (Barbe *et al*., 2009). It can now be expected that, barring the inevitable mutations that appear during propagation in laboratories [see the situation for *E. coli* (Soupene *et al*., 2003)], this final sequence corresponds to an exact sequence [International Nucleotide Sequence Database Collaboration (INSDC) AccNum AL009126.2], that does not need to be re‐sequenced. In contrast, sequence annotations inevitably keep changing as the identification of gene function improves almost on a daily basis. Some genes were actually annotation artefacts, while novel genomic objects, in particular untranslated regulatory RNAs, are being discovered on a regular basis. A few years later, it had already been relevant to associate the now exact sequence with an update of the metabolic pathways that were deciphered after analysis of the genome [INSDC AccNum AL009126.3 (Belda *et al*., 2013)]. Naturally, with the genome sequence available, as well as the new ‘omics’ approaches, discoveries establishing the function of genes previously of unknown function (there was about 2000 of those, half of the genes identified in the first report of the genome sequence) kept accumulating. Here, we report the annotation of the genome sequence at the date of 15 November 2017, twenty years after its initial version, with the inclusion of a large number of newly identified functions (including several unpublished experimentally established functions, Appendix S1) and the discovery of three dozen new genomic objects with experimentally established functions (Table 1). Taking into account the current availability of the sequences of many of its strains, we took the opportunity of the present work to explore again the natural niche of *B. subtilis* as a species (remembering that because strain 168 is a laboratory strain, it is likely to have lost some of its wild type ecological potential), as well as the nature of the genes that may be considered to characterize the species, focusing on novel entries.

**Table 1:** Novel genomic objects introduced in the present annotation of the B. subtilis 168 genome

	Label	Start	Name	Function	References
ldRNA	BSU_misc_RNA_3	119855	ldlJ	Ribosomal protein L10 leader mRNA sequence	26101249
suRNA	BSU_misc_RNA_7	486092	swaO	ATP‐, cyclic di‐AMP‐sensing riboswitch	25086507, 25086509
CDS	BSU04785	528025	cmpA	Factor allowing degradation of SpoIVA by ClpXP	26387458
suRNA	BSU_misc_RNA_65	532642	sncO	ICEBs1 mobile element: conserved small untranslated RNA	20525796, 22505685
suRNA	BSU_misc_RNA_66	559610	sncZ	No identified function: borders undefined	20525796
suRNA	BSU_misc_RNA_8	626446	aswA	Adenine riboswitch	25573585
CDS	BSU09958	1071402	sscA	Spore assembly and germination protein	21670523
CDS	BSU09959	1071613	sscB	Spore assembly and germination protein	21670523
suRNA	BSU_misc_RNA_67	1233405	roxS	Small regulatory RNA (NO regulated)	28436820
CDS	BSU12815	1348356	spoIISC	Three component toxin/antitoxin/antitoxin SpoIISABC, antitoxin C	25039482, 26300872, 27294956
Riboswitch	BSU_misc_RNA_16	1376328	guwA	Guanidinium riboswitch	28212758
Riboswitch	BSU_misc_RNA_68	1395622	swmG	Magnesium riboswitch (modest affinity)	28455443
Riboswitch	BSU_misc_RNA_87	1410633	mnrW	Manganese ion riboswitch	25794618, 25794619
Riboswitch	BSU_misc_RNA_88	1457005	gswA	Riboswitch regulating ptsGHI expression via GlcT binding	15155854, 22750856
suRNA	BSU_misc_RNA_69	1483557	fsrA	Regulatory RNA controlling iron‐dependent metabolism	24576839
suRNA	BSU_misc_RNA_70	1534070	srrA	Small regulatory RNA and messenger RNA (arginine metabolism)	27449348
CDS	BSU14629	1534120	rgpA	Regulator of GapA synthesis	27449348
CDS	BSU15140	1580622	rsmH	16S rRNA m4C1402 methyltransferase	27711192
suRNA	BSU_misc_RNA_89	1780554	surX	sigW‐dependent	23155385
CDS	BSU17845	1916955	yzzP	No identified function, present in some S. pneumoniae strains	27144405
CDS	BSU18978	2069883	bsrE	Type I toxin (BsrE/AsrE)	26940229
suRNA	BSU_misc_RNA_74	2070115	asrE	Small regulatory antitoxin RNA, toxin‐antitoxin type I system (BsrE/AsrE)	26940229
CDS	BSU19749	2146053	yoyG	Putative toxin of a type I toxin family (sporulation operon)	20156992, 21670523
fCDS	BSU20049	2160397	nrdFBc	Phage SP beta nucleoside diphosphate reductase minor subunit (C‐terminus)	23391036
fCDS	BSU20051	2161778	nrdFBn	Phage SP beta nucleoside diphosphate reductase minor subunit (N‐terminus)	23391036
suRNA	BSU_ncRNA_1	2208880	aimX	Small RNA controlling lysogeny of phage SPbeta	28099413
CDS	BSU20850	2208980	aimP	Arbitrium lysis /lysogeny regulatory peptide (GMPRGA)	28099413
CDS	BSU20860	2210154	aimR	Arbitrium peptide sensor regulator	28099413
asRNA	BSU_misc_RNA_90	2219849	apbT	Antisense RNA of Toxin SpbT	24576839
CDS	BSU21000	2219960	spbT	Toxin	24576839
suRNA	BSU_misc_RNA_91	2472880	pswI	Proline T‐box riboswitch upstream of porI	21233158
suRNA	BSU_misc_RNA_82	2773783	surF	Expressed under sporulation conditions	25790031
ldRNA	BSU_misc_RNA_43	2855915	ldlU	Ribosomal protein L21 leader mRNA sequence	27381917
CDS	BSU28475	2910746	lysCB	Beta subunit of aspartokinase II	1980002
ldRNA	BSU_misc_RNA_47	2953550	ldlT	Ribosomal protein L20 leader mRNA sequence	23611891
ldRNA	BSU_misc_RNA_93	3035589	ldsD	Ribosomal protein S4 leader mRNA sequence	23611891
asRNA	BSU_ncRNA_2	3335545	auzJ	Putative antisense RNA for YuzJ putative toxin (toxin I signature)	20156992, 21670523
suRNA	BSU_misc_RNA_94	4169919	mswM	Manganese riboswitch	25794618, 25794619

## Databases for the genome

**Figure 1:**
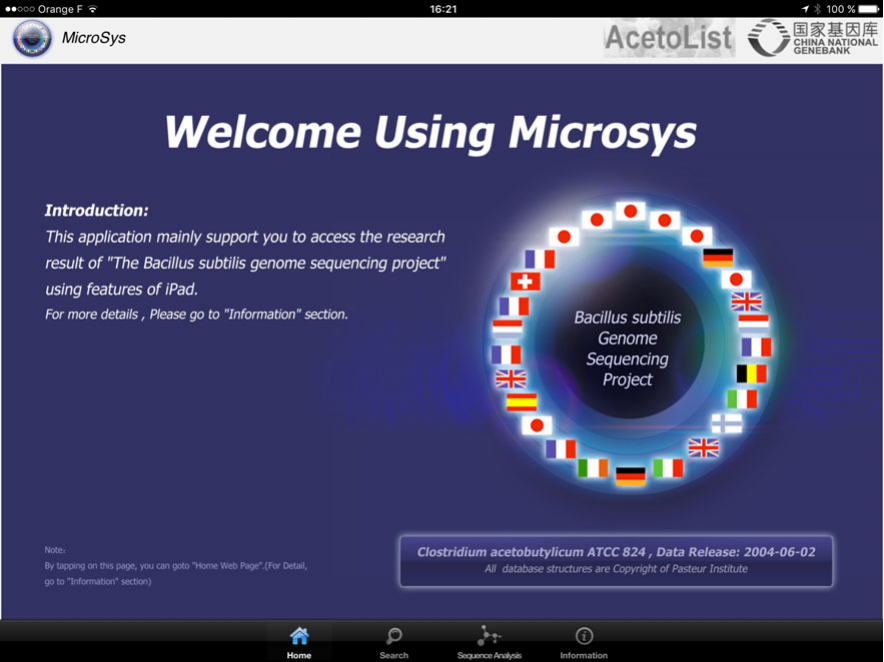
Based on SubtiList, a draft interface for microbial databases built up for tablets at the BGI.

For many years, the SubtiList database was used by most investigators as the reference database for the *B. subtilis* 168 sequence (Moszer *et al*., 2002). It was maintained at the Institut Pasteur until year 2009, when its support was discontinued. In parallel, a mirror with significant modifications (Fang *et al*., 2005) was established at the HKU‐Pasteur Research Centre Ltd where it was supported by a grant of the Hong Kong government's Innovation and Technology Commission (Biosupport) until 2010. Lack of support from the Institut Pasteur resulted in obsolescence and the Beijing Genome Institute in Shenzhen took over the baton until 2016 via the Microme Genochore microbial support. This resource, MicroSys, which had proposed a database available on tablets (Fig. 1), has since been discontinued without prior notice.

### SubtiWiki

Facing the lack of support for a facility that is of considerable interest for all investigators working with Firmicutes, Jörg Stülke and his colleagues in 2009 decided to create a Wiki site, SubtiWiki, which collates as much as possible information from the literature about the reference *B. subtilis* genome sequence (Lammers *et al*., 2010). This resource is now routinely used by the community, providing text‐based access to published information about the genes and proteins of *B. subtilis* as well as presentations of its metabolic and regulatory pathways (Michna *et al*., 2016).

### The MicroScope/MaGe platform

Annotation of genome sequences must be imbedded in knowledge generated for as many sources of information as possible. Médigue and co‐workers designed an annotation platform, MicroScope/MaGe, meant to make the most of the diverse annotations associated to bacterial genomes by imbedding in the same platform both sequence and annotation data, together with analytical methods designed to explore the data (Medigue *et al*., 2017; Vallenet *et al*., 2017). To obtain a cutting‐edge annotation of the genome of *B. subtilis* 168, we used the MicroScope platform to collect information from as many sources as possible, based on literature and extant databases. This new annotation is now available at the INSDC and at the MicroScope Website (https://www.genoscope.cns.fr/agc/microscope). Since the last update in databanks (January 2013), the annotation of about 96% of the protein‐coding genes (4097 among 4257 CDS) was revised. Furthermore, additional bibliographical references (2097 new publications among a total of 5754) were added and cover approximately 79% of the protein‐coding genes. The annotated sequence is available with the present work as Table S2.

### Tentative approaches towards a unified nomenclature

A major challenge facing genome sequence databases is gene nomenclature. Indeed, the first gene names were proposed based on phenotypes [for example in *Escherichia coli*, related to antibiotic resistance, e.g. ‘*ampC*’ for ampicillin resistance (Normark and Burman, 1977), or shape, e.g. ‘*fts*’ genes, yielding filamentation when mutated (Ricard and Hirota, 1973)]. Subsequently, names were chosen following identification of a biological function, often an enzyme function. In parallel, authors liked to propose fancy names to genes (this is well illustrated in the current gene nomenclature of *Drosophila melanogaster*). Also, genes corresponding to orthologues in different species were often named differently, depending on the inclination of the authors of the first works. Naturally, of course, many enzymes are promiscuous so that the first catalytic activity discovered in one organism could differ from that in another organism, especially when the first identifications were obtained *in vitro*. This is obviously problematical as browsing knowledge databases using gene names could help investigators to focus rapidly on their genes of interest. It is therefore recommended that the gene names of orthologues should be conserved throughout the tree of life. However, many gene products have more than one function, mediated by interactions with a variety of partners that often differ in different organisms. All this means that a fully consistent nomenclature is unlikely to be reached any time soon. Nevertheless, because having consistent names for common functions each time, a gene that has been correctly annotated would help users immensely and we have tried as much as possible to give identical names to orthologues of *E. coli* and *B. subtilis*. This was previously attempted in the GenoChore databases (Fang *et al*., 2005), where, for example, the ribosomal protein S12 gene would be named *rpsL* in all genomes, rather than use its original access tag (e.g. in *P. putida*, PP0449).

To name genes, we used an extended version of Demerec's nomenclature system (Demerec *et al*., 1968). A gene name is italicized and begins with three low case letters, followed by one or more capitals. In the best systems, there is no numeral in a wild type gene name: numerals are reserved to identifying mutant genes [e.g. *relA1* for a common mutation found in laboratory strains of *E. coli* (Harvey *et al*., 1988)]. We nevertheless still kept here the numeral 0 in sporulation genes, but we suggest that ‘0’ (*spo0A*) should soon be replaced by letter ‘O’ (*spoOA*). When genes are split into several parts, making pseudogenes, their name identifies the relevant part with a low case letter following the standard gene name [e.g. *n* for the N‐terminus and *c* for the C‐terminus, as illustrated in pseudogene *appA*, split into *appAn* (BSU11381) and *appAc* (BSU11382) in the present *B. subtilis* reference laboratory strain]. For genes identified as CDSs of unknown function, we kept the ‘*y*’ (‘Why’) nomenclature with the Demerec format, until a function could be ascribed to the gene, at which time, the name was changed into a standard gene name, preferably using the name proposed by the authors that identified the function, when it existed. When renaming, we kept the last capital letter of the ‘y’ name in the final name when this did not create duplicated names (e.g. *skiX* replaces *yknX*). We finally noticed that investigators often explore the literature and databases using a gene name. It is therefore highly inconvenient when a gene name corresponds to a common English word (*e.g. hinT* or *thiS*). We therefore tried as far as possible to avoid such common spelling when creating new names, and we recommend, for future annotations, to try and replace those unwieldy names by new ones (a general possibility is to use the extended Demerec's rule, adding a second letter after the final capitalized letter of the gene name). Because many genes have a variety of names in the literature, synonyms were included in the gene data file, which is indexed using a unique accession number [e.g. *thiO*, with synonyms *yjbR* and *goxB*, AccNum BSU11670 label, codes for a promiscuous glycine oxidase that is involved in the first step of thiamine biosynthesis (Jurgenson *et al*., 2009)].

## *Bacillus subtilis* in 2017

Experimentally‐rooted database curation is essentially manual, and therefore considerably time‐consuming. Here, data from the literature were systematically collected by exploring PubMed, PubMed Central and SubtiWiki, and browsing the Internet with ‘*y*’ gene names as keywords as sources of information. In addition, we used a functional analysis approach of the type that is fruitful when trying to construct relevant chassis in synthetic biology [SynBio (Harwood *et al*., 2013)]. This entails considering cells as computers making computers, with all the relevant prerequisites (Danchin, 2009).

### Making inferences using synthetic biology approaches

**Figure 2:**
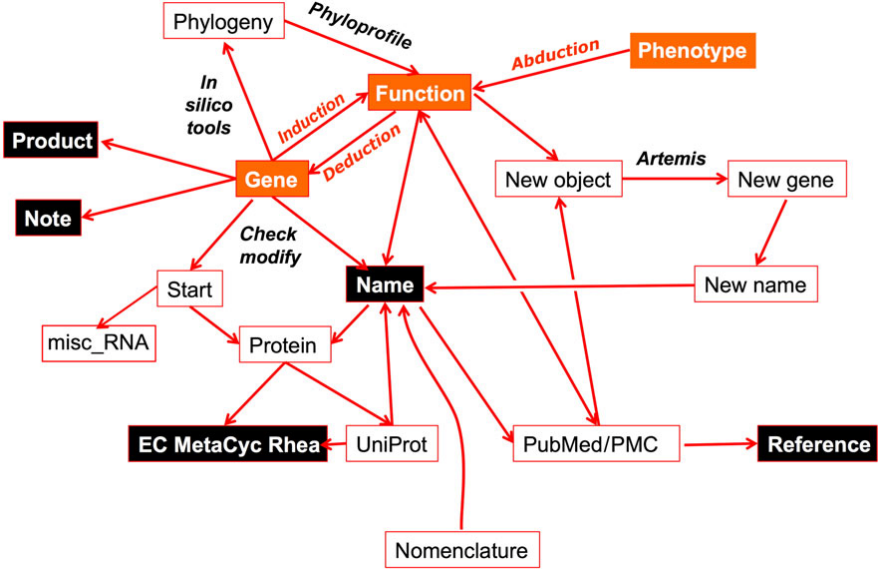
Scenarios for annotation. Annotation combines three approaches: data‐, hypothesis‐ and context‐driven. The first one is based on induction, the second on deduction and the third on abduction, combining functional, phenotypic and sequence data (orange boxes and see text). The outcome of the procedure results in the identification of a gene product, a gene name, participation in metabolic reactions and literature references identified by PubMed identifiers (black boxes). Free text notes are also provided to help understanding the biologically relevant context of each particular gene.

Function identification can be derived in three major ways (see Fig. 2 for a general scenario used here for genome sequence annotation). First, a bottom‐up approach uses alignments of sequences with proteins of experimentally known function (this is the standard approach). Second, symmetrically, a top‐down complementary approach that follows the trend developed in SynBio studies with emphasis on the machine reading the programme [the ‘chassis’ in the relevant jargon (de Lorenzo and Danchin, 2008)]. It starts from building up a functional partition of the genome into two master functions: 1/functions (hence genes) required for constructing a progeny; 2/functions required for occupying a specific niche and functions used to create specialized devices – cell types or organelles – meant to explore the environment. Finally, an abductive reasoning approach rests on educated guesses that explore the consequences of specific predictions (‘shot in the dark’: facing a forest at night, fire, and if something cries, look for it; if not, try again). An example of this situation is reflected in the discovery of the unexpected pathway allowing the organism to use S‐methyl‐cysteine (SMeC) as a sulphur source. Knowing that dioxygen was involved, the expected pathway was predicted as an oxidation step of the sulphur atom. After a long series of unsuccessful approaches, it was observed that a DefB mutant of strain 168, lacking one of the two amino acid deformylases of strain 168, did not grow on SMeC. This triggered the hypothesis that the methyl‐group was oxidized rather than the sulphur atom. This allowed deciphering of the entire pathway (Chan *et al*., 2014).

The cell‐as‐a‐computer model splits functions into two major types. Those which run the system [equivalent to the operating system (OS) of a computer] and those which use the cell for specific purposes (equivalent to the applications run by the computer). The former are limited in number. They are those identified in the minimal genomes constructed for SynBio approaches. We named this set the paleome (Acevedo‐Rocha *et al*., 2013). It comprises the core translation, transcription and replications machineries, together with basic membrane functions involved in waste disposal, basic ion supply, in energy generation and cell wall synthesis, as well as a key set of central metabolism enzymes. Remarkably, exactly as for authentic computers, a few paleome functions are specific to a particular clade, as OS functions may be specific to a particular computer brand. An illustration of such inevitable kludges required to implement an abstract schema into a material world is, in the Firmicutes/Tenericutes clade, the requirement for a protease that splits off the first nine residues of ribosomal protein L27 initially used as a scaffold, after assembly of the ribosome (Danchin and Fang, 2016). In the present annotation, it was found *in silico* that gene *rppA*(*ysxB*) of the *B. subtilis* genome codes for this function (Wall *et al*., 2017). As a rule, we further substantiated our predictions, when not directly based on experiments in *B. subtilis*, by conservation of essential amino acid residues or specific neighbourhoods, provided by synteny or by co‐evolution profile (or both). Another feature of the process of translation has also been revived with the identification of an important role of formylation of the methionine residue loaded on initiator tRNA (Cai *et al*., 2017). This revives an open question about the apparent redundant role of formylation in translation previously explored in *E. coli* where an allosteric modulation of the 70S ribosome structure may shift back to initiation in polycistronic operons without a requirement for the dissociation of ribosomal subunits during the translation of contiguous cistrons (Petersen *et al*., 1976; Yamamoto *et al*., 2016).

In the domain of replication, the co‐evolution profile of DNA polymerase III alpha subunits (Engelen *et al*., 2012) contributed fruitfully to the present annotation. It allowed us to identify several important functions specific to the *B. subtilis* species. In the same way, the degradosome structure of Firmicutes is different from that of Proteobacteria, for example, and quite consistent. In particular, degradation of messenger RNAs involves the combined activities of endonucleases, 3′‐end exonucleases and 5′‐end exonucleases, with a specific set of enzymes that have both activities identified experimentally in *B. subtilis* RnjA and RnjB. The exact function of the latter will need to be further characterized (Gao *et al*., 2017) as it seems to be present even in streamlined Tenericutes (Hutchison *et al*., 2016), while it co‐evolves mainly with genes of unknown functions (Engelen *et al*., 2012). The set of persistent genes identified in Firmicutes defines the *B. subtilis* paleome. The function of most of the genes of this basic OS has now been identified. Table S1 summarizes the most recent functional identification of the genes that have long remained without an ascribed function, in parallel with the streamlined paleome functional set.

### Strain 168 among other *B. subtilis* strains

Sequencing genomes has become much simpler and cheaper since the date of publication of the sequence of strain 168. While it remained the only *B. subtilis* genome available for many years, there has been a significant effort to sequence other strains in the last decade. This resulted in more than 45 completely sequenced genomes by the end of 2016, and around one hundred high‐quality draft genomes are deposited in the NCBI RefSeq database (ftp://ftp.ncbi.nih.gov/genomes/refseq/bacteria/Bacillus_subtilis/). The number of complete genomes is likely to increase further in the near future thanks to long‐read sequencing technologies. The availability of genomes for many strains opens up new avenues for research. Most importantly, it opens up the possibility of using population genomics data to make inferences about the function of genes in the genome and their ecological role. Yet before making such analyses, one must draw a line between authentic genomes of *B. subtilis* and those of other highly similar species. Currently, and this taxonomic misannotation is unfortunate, several complete genomes labelled as *B. subtilis* are genetically quite distant from the reference strain, with an average nucleotide identity (ANI) lower than the proposed minimal threshold for defining the species [94% (Konstantinidis *et al*., 2006; Richter and Rossello‐Mora, 2009)]. While the definition of bacterial species has been based essentially on physiological and biochemical traits, recent works suggest that population genetics and ecological definitions might provide more meaningful definitions of species (Gevers *et al*., 2005; Ward *et al*., 2008).

**Figure 3:**
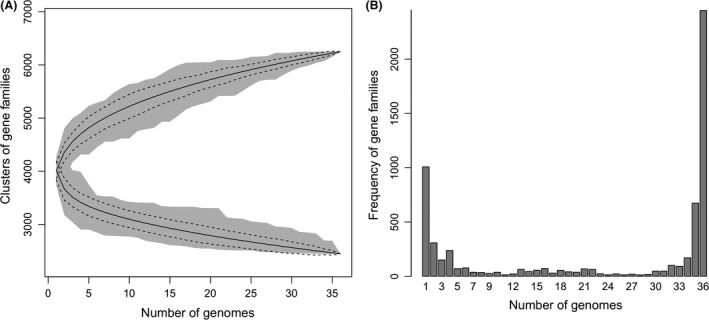
Analysis of protein‐coding genes in 36 complete genomes of *B. subtilis*.A. The core and pan‐genomes were computed for random samples of increasing size of the 36 genomes. The shaded regions indicate the range of variation of these values.B. The frequency of the presence of each gene family from those that are present in only one strain (peak at 1) to those that are components of the core genome (peak at 36). The identification of the families of core and pan‐genomes followed the methodology of (Touchon *et al*., 2014).

We analysed the diversity of protein‐coding gene repertoires of 36 complete genomes of *B. subtilis* (selected after using the threshold of ANI > 94% relative to strain 168). The absolute numbers provided by these analyses must be handled with care, as they depend on the methods used and on the homogeneity of the annotations. To ensure that annotations are as homogeneous as possible, we used the re‐annotations of RefSeq (even though, for strain 168, they are not the best ones). The core genome, that is the set of genes present in all strains, was composed of around 2500 genes (Fig. 3A). This value is still slightly decreasing with the increasing number of sequenced genomes, suggesting that it may be even smaller. However, some of the decrease in the core genome with increased sampling may be due to recent deleterious mutations, yet to be purged by natural selection, or to annotation or sequencing errors. Hence, it is more meaningful at this stage to mention that 3291 genes families are present in more than 95% of the strains. These account for around three‐quarters of the genome of strain 168. The pan‐genome, the diversity of different gene families encountered in the set of the 36 genomes in the species, is much larger, reaching ~6250 genes, about 50% more than the gene repertoire of the average genome. Sampling more genomes will certainly increase this number, given the shape of the cumulative curve (Fig. 3A), and as about 1000 gene families are only found in one strain (Fig. 3B). Matching many other bacterial genomes (Touchon *et al*., 2009; Collins and Higgs, 2012), the majority of gene families are present in either very few, or most genomes of the species.

### The genome sequence highlights the *B. subtilis* ecological niche

This study demonstrates that a core of approximately 2500 genes reflects the gene setup of *B. subtilis* as a species. It comprises a paleome, described previously, that is essentially shared with that of the minimal Firmicute/Tenericute genome, made of <500 genes (Danchin and Fang, 2016), associated to a complement that defines the minimal niche occupied by this species, its species‐specific cenome – its genes coding for context‐specific functions (Acevedo‐Rocha *et al*., 2013). Most genes of the paleome have a well‐characterized function. We noticed that several engineer‐type structural functions linked to the building up of the ribosome nanomachine as well as other important RNA structures such as riboswitches are now well understood: RulR(YlxR) codes for molecular ruler (Zhang and Ferre‐D'Amare, 2016), and KtuQ(YlxQ, RpmXA) and KtuS (YbaB, RpmXB) are RNA‐binding proteins specifically associated to kink turns (Huang and Lilley, 2016). Once identified, the association of the functions coded in the cenome match well with the conditions of the first isolation of *B. subtilis* in the wild, as reported in the Bergey manual (Sneath, 1986); ‘hay, or grass bacillus’ in English, ‘laseczka sienna’ in Polish, ‘kusa no saikin’ in Japanese (*B. subtilis* enriched from rice straw is used to make the popular soy beans fermented food natto). This also reminds us of the Pasteur/Pouchet controversy about the origin of life (Roll‐Hansen, 1979): Pouchet boiled hay extracts as a way to ‘sterilize’ growth media, and because spores resisted he could antagonize Pasteur. All these experiments point to this bacterium as tightly associated to herbaceous plants, both in the rhizosphere and in the phylloplane. *Bacillus subtilis* strains have even been found as beneficial endophytic bacteria in a variety of plants (Gond *et al*., 2015; Ding *et al*., 2017). A significant number of genes are indeed explicitly involved in direct interaction with plants [e.g. roots (Habib *et al*., 2017), or leaves (Zeriouh *et al*., 2014)], either positively or as scavengers of metabolites such as rhamnosides from decaying plants. As a case in point, among many other examples, YfmS, a chemotaxis sensory transducer recognizing a still unknown substrate is involved in the colonization of *Arabidopsis thaliana* roots (Allard‐Massicotte *et al*., 2017). Last, the Spo0A protein that controls the fate of cells as vegetative, spores or biofilm‐forming cells, is key to root colonization (Grau *et al*., 2015). Strain 168, however, is mutated in several genes that would compromise its occupation of this natural biotope: it requires tryptophan for growth because *trpC* has been inactivated by a frameshift, and, in the same way, it cannot properly colonize roots because of inactivation of gene *sfpA* for example. However, strain OKB105, which is a derivative with an intact *sfpA* gene, is able to produce non‐ribosomal peptides and polyketides restoring its authentic plant interaction (Xie *et al*., 2014).

Finally, and this is remarkable, *B. subtilis* possesses a blue light receptor, BlrA (formerly YtvA) related to plant phototropins, coupled to a transcriptional complex that monitors the presence of light in the environment. Besides being sensitive to light, this receptor senses the presence of oxygen (Losi *et al*., 2002). It carries a LOV (light, oxygen and voltage) domain and a STAS (sulphate transporters and antisigma‐factor antagonists) domain and binds FMN (flavin mononucleotide) as a chromophore. Its cycle of activation by light/recovery is also tuned to the environment by features such as hydration (Pennacchietti *et al*., 2014). Sensing light is a way for cells to tell immediately where they are located. Interestingly, *Klebsiella pneumoniae*, which, contrary to what its name would suggest, has a plant niche somewhat similar to that of *B. subtilis*, possesses a putative receptor NifL [involved in oxygen regulation of nitrogenase synthesis, binding flavin nucleotides (Christie *et al*., 1999)] that has features in common with those of BlrA. *Photorhabdus luminescens* also codes for a protein, Plu4388, with a domain that may monitor light, perhaps allowing light communication between these photon producing cells. Even *Pseudomonas putida* PP_4629 protein may be a photoreceptor. In *B. subtilis,* this is consistent with a plant niche alternating between the phylloplane (light, dioxygen and dry conditions) and the rhizosphere (dark, low oxygen and humidity). In terms of coupling light with environment‐dependent gene expression, this also fits well with another functionally convergent light‐sensing system discovered in *E. coli*, where the light‐sensitive BluR transcriptional regulator couples the response to light oxygen and temperature [*E. coli* cycles between a warm dark anaerobic environment and a cool aerobic environment (Tschowri *et al*., 2012)].

Beside these widespread genes, *B. subtilis* displays specific features involving cell differentiation, on the one hand via sporulation and motility organelles, or via formation of multicellular entities, biofilms; on the other hand it also encodes phages or phage remnants. The corresponding set of genes that we may name histome (from ἱστος, tissue) comprises an appreciable part of the genome [more than 300 genes for sporulation, and 51 for appendages, flagella (43) and pili (8), often grouped into islands].

The role of phages has also to be revisited. Temperate phages were long considered to be in a dormant state, waking up in specific conditions of the environment. Yet, phage induction is involved in a variety of differentiation processes. In strain 168, the skin element, for example, is removed in the mother cell during the sporulation process, generating the sporulation‐specific sigma factor K (Krogh *et al*., 1996). In the same way, the *spsM* gene is interrupted by bacteriophage SPbeta which is excised during the sporulation process using two phage‐encoded proteins, SprA and SprB (Abe *et al*., 2017). This is now recognized as a new role of lysogeny, named ‘active lysogeny’, that provides yet another account for the presence of bacteriophages within bacteria (Feiner *et al*., 2015), with *B. subtilis* as a paradigmatic example.

### Novel features extracted for the genome sequence reannotation

In the present release of the *B. subtliis* 168 reference sequence annotation, we have included some new genomic objects, in particular RNAs, when this was linked to identified functions (we still left aside much of the many transcripts identified via RNAseq sequencing but not explicitly linked to identified functions) and some protein‐coding genes, such as *spoIISC* coding for the third element of the three‐components toxin/antitoxin/antitoxin SpoIIS sysem [Table 1, (Gabrisko and Barak, 2016)]. We have also experimentally authenticated genes such as the transporters of methylthioribose [MtrA(previously YfnA) for influx and MtrE(B/Y) for efflux, and the ribose transporter, see Appendix S1] and an aminotransferase DapX (previously PatA), required for an essential step in lysine biosynthesis proceeding via an acetylated intermediate, as also does the MetAA intermediate, in contrast to the situation in *E. coli* [Appendix S1 and see (Bastard *et al*., 2017)].

We further focused on specific metabolic features that have until recently been overlooked. Bacteria must cope with inevitable errors of metabolism (Danchin, 2017), mediated in particular by a list of expected toxic side reactions (Lerma‐Ortiz *et al*., 2016). The *B. subtilis* metabolic setup illustrates variations upon this very general theme. For example, a large variety of organisms use glutathione as a general detoxifying compound. In contrast, in *B. subtilis,* glutathione is replaced by a counterpart, bacillithiol, that plays most if not all of the roles discovered previously for glutathione (Chandrangsu *et al*., 2017b). Interestingly, the enzymes that use bacillithiol are often counterparts of enzymes identified elsewhere, but are not true orthologues as they must accommodate a different thiol substrate. A similar situation is observed in Actinomycetes, where mycothiol replaces glutathione (Rawat and Av‐Gay, 2007). This is a very important observation that should be taken into account when considering clusters of orthologues. Metabolic accidents contribute to ageing, in particular via synthesis of dicarbonyls such as methylglyoxal (MGO) or fumarate (Danchin, 2017). The latter reacts with cysteine in proteins or glutathione, forming S‐(2‐succinyl) cysteine inducing senescence in animals (Miglio *et al*., 2016). MGO results from the action of MGO synthase, MgsA, the function of which is still a matter of speculation (Danchin, 2017). *Bacillus subtilis* has an arsenal of genes that allows it to cope with this toxic molecule [AkrN(YhdN) aldo/keto reductase specific for NADPH; KhtSTU (YhaSTU) proton/potassium antiporter (Chandrangsu *et al*., 2013); SufL (YraA) deglycase, a general stress protecting enzyme (Abdallah *et al*., 2016); GlxB(YurT) methylglyoxalase, lactoylbacillithiol lyase and YvgN promiscuous glyoxal/methylglyoxal reductase, several of them involving bacillithiol directly or indirectly via controlling potassium transport].

Other types of errors result from the presence of mimics of authentic functional metabolites and this must be remedied. As a case in point PgeF (YlmD, EcYfiH), a factor involved in maintaining the composition of the murein peptides complements an *E. coli yfiH* defect. Lack of PgeF results in the incorporation into the PG sacculi of non‐canonical amino acids, L‐serine or glycine in place of L‐alanine (Parveen and Reddy, 2017). Among widespread sources of metabolic errors, non‐proteinogenic amino acids should be prevented from entering the translation process, and a variety of pathways cope with this situation. A general feature of the metabolic processes that deal with analogues of authentic functional metabolites is similar to that found in a chemist's laboratory: protection (N‐acetylation of the unwanted aminoacid) to prevent hazardous reactions, followed by deprotection at the end of the inactivation pathways (Chan *et al*., 2014). The large collection of N‐acyl‐transferase genes present in the genome (46 genes) and often with no identified function should be explored for this type of function. Among those are also safeguard systems that protect residues within proteins (usually lysine residues, but also arginine or histidine residues) against spurious modification by reactive metabolic intermediates (Kim *et al*., 2013). Interestingly, as is commonplace in evolution processes, once a programmed modification exists, it can be recruited for further functions, in particular regulatory functions (Kosono *et al*., 2015). Coenzymes are prone to accidents: NAD(P)H is hydrated into an analogue that would clog many pathways if it were not converted back to the active form by NnrA(YxkO), a repair enzyme (Petrovova *et al*., 2014). S‐adenosylmethionine [(S,S)‐AdoMet] may isomerise at the sulfonium atom and the accidental isomer (R,S)‐AdoMet has presumably found a way to remain a methyl‐donor via an homoscysteine methylase using both isomers [a domain in SamT (Lu *et al*., 2010), and possibly YbgG, similar to *S. cerevisiae* methyltransferases Mht1 and Sam4 which could also be a much needed AdoMet racemase (Vinci and Clarke, 2010)]

In the same way, while iron is essential in many processes (in particular in respiration), *B. subtilis* has an interesting preference for manganese [see (Chandrangsu *et al*., 2017a)], for example with two transporters, a major one MneP(YdfM) and MneS(YeaB) and a minor one (Huang *et al*., 2016). This may explain why iron is dispensable from a variety of Firmicutes (mostly Lactobacilli (Weinberg, 1997)) and the derived clade of Tenericutes (Danchin and Fang, 2016). Finally, it is important to stress in this update that *B. subtilis* harbours a new regulator, cyclic diAMP, the main function of which, potassium homoeostasis, has been deciphered by Jörg Stülke and his co‐workers (Gundlach *et al*., 2017).

## *Bacillus subtilis* exploring its environment

In the previous paragraphs, we have described the behaviour of *B. subtilis* in its preferred environment as revealed by the present genome annotation update. Several additional features were also revealed during this undertaking. Ecological niches keep changing and bacteria must accommodate to new and often hostile environments, while trying to stick to those environments that evolution has directed them to favour. Three major functions are linked to this situation: overcoming deleterious actions of non‐living and living organisms, escaping to other niches, possibly far away or staying in place. *Bacillus subtilis* monitors this situation via specific sigma factors (Helmann, 2016) and protein phosphorylation cascades (Schultz, 2016; Pane‐Farre *et al*., 2017). These will not be further discussed here (except to note that the concept of stress being very ambiguous, as all living organisms suffer multiple transitions, it should probably be avoided to be replaced by the idea of transition management).

### Resisting poisons and hostile conditions

Among interesting features, recently identified in the genome is a heteromeric transporter CrcBA CrcBB, allowing resistance to fluoride ions (Ji *et al*., 2014; Macdonald and Stockbridge, 2017). Indeed, it has been found that fluoride flooding has happened repeatedly (volcanic ashes, local environments and rock weathering) resulting in an average concentration of 625 mg kg^−1^ in different rock types (Tavener and Clark, 2006) and diffusing into plants. In parallel, GswA, a member of a riboswitch family long of unknown function, has been functionally identified as a result of its ability to bind guanidine (Lilley, 2017), and control expression of a guanidinium exporter, GndCD (YkkCD). A variety of quorum‐sensing systems exists in *B. subtilis*, with a novel one involving kanosamine, a metabolite that also acts as an antibiotic against a variety of microbes (van Straaten *et al*., 2013; Tojo *et al*., 2014; Vetter and Palmer, 2017). Finally, *B. subtilis* is able to scavenge complex molecules made by other organisms, such as the xenosiderophore schizokinen via the specific transporter SxzYZA (Podkowa *et al*., 2014).

The plant environment suffers alternating dry and wet conditions and this results in considerable changes in osmotic pressure, monitored by mechanosensing (Belas, 2014). *Bacillus subtilis* codes for at least five such safety valves (McsC, McsL, McsT, McsY), one specific for sporulation (SpoVAC) that open up upon lethal increase in osmotic pressure. Some of those may also leak out or in antibiotics, leading to constitutive resistance (Song *et al*., 2013) or sensitivity (Jiafeng *et al*., 2015).

### Moving around

Swimming in liquid media and swarming on surfaces are two major motile behaviours of bacteria. Swimming bacteria use chemotaxis to find nutrients and avoid toxic environments. By contrast, swarming bacteria suppress chemotaxis and self‐organize in a collective motion to explore novel niches while being protected by a mass effect (Harshey and Partridge, 2015). Remarkably, swarming appears to be dependent on a modification of translation factor EF‐P by a 5‐aminopentanol group, as swarming is defective in the absence of EfpI(YmfI) that reduces aminopentanone to aminopentanol (Hummels *et al*., 2017). In addition, *B. subtilis*, even when devoid of appendages, is capable of sliding on surfaces (Kovacs *et al*., 2017), dependent on the presence of surfactin (defective in strain 168, due to pseudogene *sfpA*) and of exopolysaccharides discussed below, that, interestingly, generate osmotic pressure in the extracellular space (Grau *et al*., 2015).

### Making biofilms

Exploration requires moving around, but when conditions are stably profitable, it is advantageous to find a way to stay around. This is illustrated by yet another case of convergent functional evolution, where many species of bacteria evolved a variety of mechanisms to structure sessile biofilm communities. *Bacillus subtilis* biofilms display complex architectures that, again, adapt to the plant world, with alternating dry and wet conditions. Cells are encased within a polysaccharide complex made of exopolysaccharides secreted by the bacteria (Hobley *et al*., 2015). The polyamine spermidine activates matrix synthesis via expression of regulator SlrR (Hobley *et al*., 2017).

Further exploration of the metabolism of inositol, identified, as expected for catabolism, an NAD‐dependent dehydrogenase IolX. Intriguingly, two dehydrogenases, IolU(YulF) and IolW, associated to a presumably anabolic process because they are NADP‐dependent (Kang *et al*., 2017), are possibly involved in biofilm formation [inactivation of the counterpart of IolU generates a biofilm defect in *Streptococcus mutans* (Yoshida and Kuramitsu, 2002)]. FbnA(YloA) is another protein that is likely to be involved in cell adherence to a variety of substrates and belongs to the biofilms’ setup [deficient cells are deficient in biofilm (Rodriguez Ayala *et al*., 2017)]. Biofilms form highly hydrophobic communities that resist wetting but also solvents and biocides. Hydrophobicity is essentially caused by secreted protein BslA with a small contribution of BslB(YweA), its paralogue (Morris *et al*., 2017), via production of a leaf/petal‐like hydrophobic behaviour (Werb *et al*., 2017). In the biofilm, synthesis of BslA is tightly regulated and the resultant protein is secreted into the extracellular environment where it forms a barrier allowing the *B. subtilis* cells to shelter under a ‘protein raincoat’ (Arnaouteli *et al*., 2016).

### Seeding the earth with a progeny

Plants, which cannot move, nevertheless colonized a considerable area of the Earth. This is because they produce seeds, which carry over their genome using a huge number of processes to escape far from their origin. The same is true for bacteria that make spores, specific structures that can sustain hardships and then germinate when conditions appear to be proper to sustain life. Sporulation, indeed, has been a major research topic for *B. subtilis* studies, providing models that are used ubiquitously to account for the process in a variety of microbes (Huang and Hull, 2017). The vast majority of our knowledge on *B. subtilis* sporulation was described in previous updates [in particular with a progressively increasing number of sporulation genes since the early times of genetic analyses (Piggot, 1973)], and we will only point out a recent observation related to this interesting process. Sporulation is costly (it requires the death of a mother cell) and the decision process to choose between other differentiated states of the bacteria is therefore of the utmost importance. Many of the components of the decision‐making machinery have been identified (Decker and Ramamurthi, 2017), most of them converging to protein Spo0A (Dubnau *et al*., 2016), which appears to be the hub at which the various stages of *B. subtilis* development are decided. In fact, the exact role of the phosphorylation cascades separating information channels (signal transduction) in cells remains open. As an example, the previously proposed notion that the NAD+/NADH ratio controls the major sporulation kinase KinA activity through the PAS‐A domain of the enzyme has been refuted (Kiehler *et al*., 2017), opening up again the question of the signals that trigger developmental processes in *B. subtilis*. These information channels are mediated by histidine kinases that have common properties, but nevertheless can channel information along highly specific pathways (Abriata *et al*., 2017) avoiding parasitic cross‐talk (Laub, 2016).

## Conclusions

Genome annotation is a way to progressively build up a consistent picture of the manner in which living organisms develop in a particular niche. While back in 1991, well before the genome sequence was completed, the acquisition of newly sequenced large genome contigs revealed that half of the putative genes thus identified were unknown both in structure and in function (they were then named elusive, esoteric, conspicuous – EEC – genes by Piotr Slonimski at a meeting in Elounda, in Greece) their role is progressively revealed owing to the hard work of investigators all over the world. Many still remain to be deciphered, and this will often bring about new concepts, such as the CRISPR‐Cas phage immunity system (absent from *B. subtilis* 168), new structures (such as K‐turn RNA‐binding proteins) or new chemical processes (such as the requirement for a protection/deprotection cycle to cope with close analogues of authentic cell building blocks). We hope that, in addition to the new knowledge that will spread to the community, this type of work will attract young investigators to follow through and take over the helm.

## Authors’ contributions

AD organized this work and wrote the bulk of the article, to which all authors contributed. He annotated all genes using the MaGe/Microscope platform, maintained by CM and DV. RB focused on annotation of plant‐related genes and genes involved in secondary metabolism. CRH adapted the text to a large audience of microbiologists. EPCR performed the *in silico* analyses of strains of *B. subtilis* related to strain 168 and wrote the corresponding section. AS focused on annotation of sulfur‐related genes and performed experiments to close gaps in metabolic pathways. DV prepared the final annotation table and deposited it at the ENA‐INSDC archive.

## Conflict of interest

None declared.

## Supporting information


**Table S1.** The extended *Bacillus subtilis* paleome

**Table S2.** *Bacillus subtilis* 168 annotated genome in the EMBL‐ENA format.

**Appendix S1.** Experimental identification of methylthioribose transport and a missing step in lysine biosynthesis.
