# Supplementary material for: Bacillus subtilis, the model Gram‐positive bacterium: 20 years of annotation refinement
Source: Microb Biotechnol. 2017 Dec 26;11(1):3–17. doi: 10.1111/1751-7915.13043 (PMC5743806; doi:10.1111/1751-7915.13043)
Supplement: Supplementary file 3 — Appendix S1. Experimental identification of methylthioribose transport and a missing step in lysine biosynthesis. [file MBT2-11-3-s003.doc]

**Supplementary text to**

**Bacillus subtilis, the model Gram-positive bacterium: twenty years of annotation refinement**

Rainer Borriss, Antoine Danchin, Colin R Harwood, Claudine Médigue, Eduardo PC Rocha, Agnieszka Sekowska, David Vallenet

by Agnieszka Sekowska

Here we identified experimentally some key functions that will help investigators to better their knowledge of B. subtilis. This is illustrated by the identification of methylthioribose transporters as well as identification of a cardinal missing enzyme in lysine biosynthesis, N-acetyl-L,L-diaminopimelate aminotransferase.

**Transport of methylthioribose**

Bacillus subtilis has a complete methionine salvage pathway (MSP) that allows the salvage of methionine when recycling S-adenosylmethionine derivatives (Sekowska et al., 2004; Albers, 2009). Methionine is a metabolite in short supply in the environment of B. subtilis. Salvage is important and the pathway is driven forward by an aminotransferase that uses glutamine instead of glutamate as an alpha-amino group donor, followed by the action of an omega-amidase, MtnU, ensuring that methionine is not degraded (Belda et al., 2013). In this pathway, 5-methylthioribose (MTR) is produced by a promiscuous nucleosidase, MtnN, then phosphorylated using an MTR kinase (EC 2.7.1.100), suggesting that the bacteria may also use MTR produced by other sources when sulfur availability is limiting. This enzyme is present in a variety of organisms that are limited in sulfur supply (Sekowska et al., 2001; Sun et al., 2004). To initiate this pathway MTR must first be imported into the cell. Nothing being known about MTR transport systems, we endeavoured to identify them in B. subtilis, known to code for MTR kinase (MtnK) and therefore expected to be able to metabolise the molecule. This allowed us to identify several such transporters, belonging to permease families of previously unknown function.

**Results**

Identification of genes coding for transport of methylthioribose

When unravelling the methionine salvage pathway using a transposon library to generate mutants in B. subtilis, we identified mutants resistant to trifluoromethylthioribose (3FMTR) (Sekowska and Danchin, 2002), an analog of MTR toxic when metabolised (Gianotti et al., 1990). This allowed us to identify the region involved in MTR metabolism. Unfortunately, using this screening procedure we failed to uncover inactivated genes coding for MTR transporters. This was not unexpected, as it is repeatedly observed that metabolites are transported via multiple permeases, with different levels of sensitivity and specificity.

Thus, an in vivo screening did not readily identify the target genes we were looking for. We therefore resorted to comparative genomics to explore the context of the MSP genes in a variety of bacterial genomes. Several genome sequences carried clusters of genes comprising transporter genes. In particular, in the small genome of the plant pathogen Xylella fastidiosa, the operon coding for MtnA and MtnK also harbours two genes coding for closely similar permease subunits of a putative ABC transporter (XF2207 and XF2208). Interestingly, BLASTP searches showed that there is a counterpart of both XF2207 and XF2208 in many bacterial species, spanning a large part of the tree of life. In contrast, they are absent from E. coli K12 [as expected for bacteria exporting MTR, not metabolising it further (Schroeder et al., 1972)], but present in E. coli S88 (which codes for MtnA and MtnB and must therefore display some sort of MTR metabolism). Bacillus subtilis is a good candidate where to look for a MTR permease as we found that wild type B. subtilis is able to use MTR as its sole sulphur source, thus demonstrating that the compound must be readily transported into the cell. Interestingly, there is a counterpart of the Xylella transporters in B. subtilis, transporter YfnA, annotated as a putative transporter of the multi-membrane-spanning class belonging to the cationic amino acid transporter APC superfamily.

Comparing growth of a mutant where gene yfnA was disrupted (strain YFNAd) with that of the wild type showed that its inactivation established almost full resistance to 3FMTR at 37°C (Figure 1).


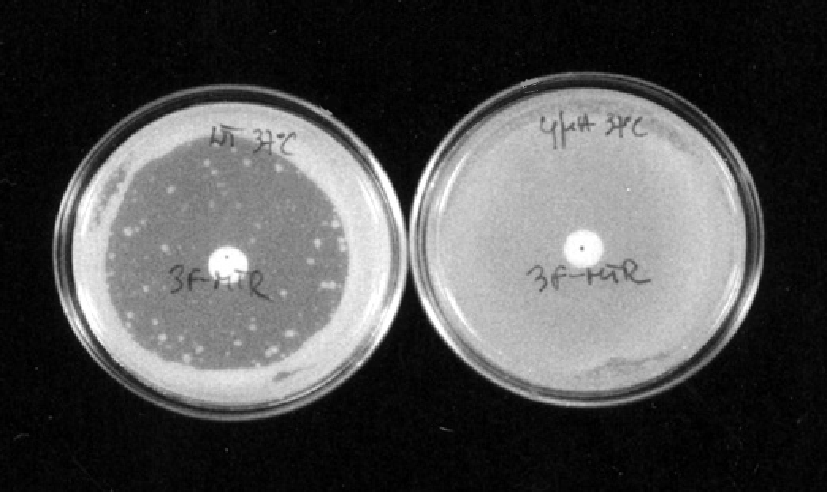


**Figure 1**. The sensitivity to 3FMTR of B.subtilis 168 WT and YFNAd strains at 37°C.

This demonstrated that permease YfnA (now MtrA) is key to MTR entry into the cell. At 46°C the strain remained sensitive to the toxic compound but less so when compared to the wild-type parent strain, indicating that at least one other permease can transport MTR.

Secondary MTR transporters

Beside its main catabolic pathway, ribose can be metabolised in B. subtilis via enzymes of the MSP (Nakano et al., 2013). We expected that the ribose metabolic pathway, encoded by the rbsDACB operon that comprise genes for an ABC transporter, may also code for a MTR transporter. We constructed a double mutant yfnA ΔrbsDACB. The mutant strain was fully resistant to 3FMTR at 37°C and significantly more resistant than the yfnA parent at 46°C (not shown), demonstrating that the ribose transport system is able to transport MTR also, albeit with lower affinity as compared to MtrA. Two other candidate transporters were subsequently explored: a guanosine transporter (NupQ) and a paralog of MtrA (YhdG). The triple mutant yfnA ΔrbsDACB nupQ (previously yufQ) was fully resistant to 3FMTR at 37°C and almost completely at 46°C showing that some MTR transport is mediated by NupQ.

To substantiate the role of these transporters in permeation of authentic MTR we grew these mutants on MTR as sole sulphur source. The mutant yfnA and the double mutant yfnA ΔrbsDACB displayed normal growth on MTR (1 mM concentration) showing that MTR could permeate the cell at a rate sufficient to allow normal sulphur supply. By contrast, the triple mutant yfnA ΔrbsDACB nupQ grew poorly on MTR at low concentrations (1 and 5 µM) (Fig.2).


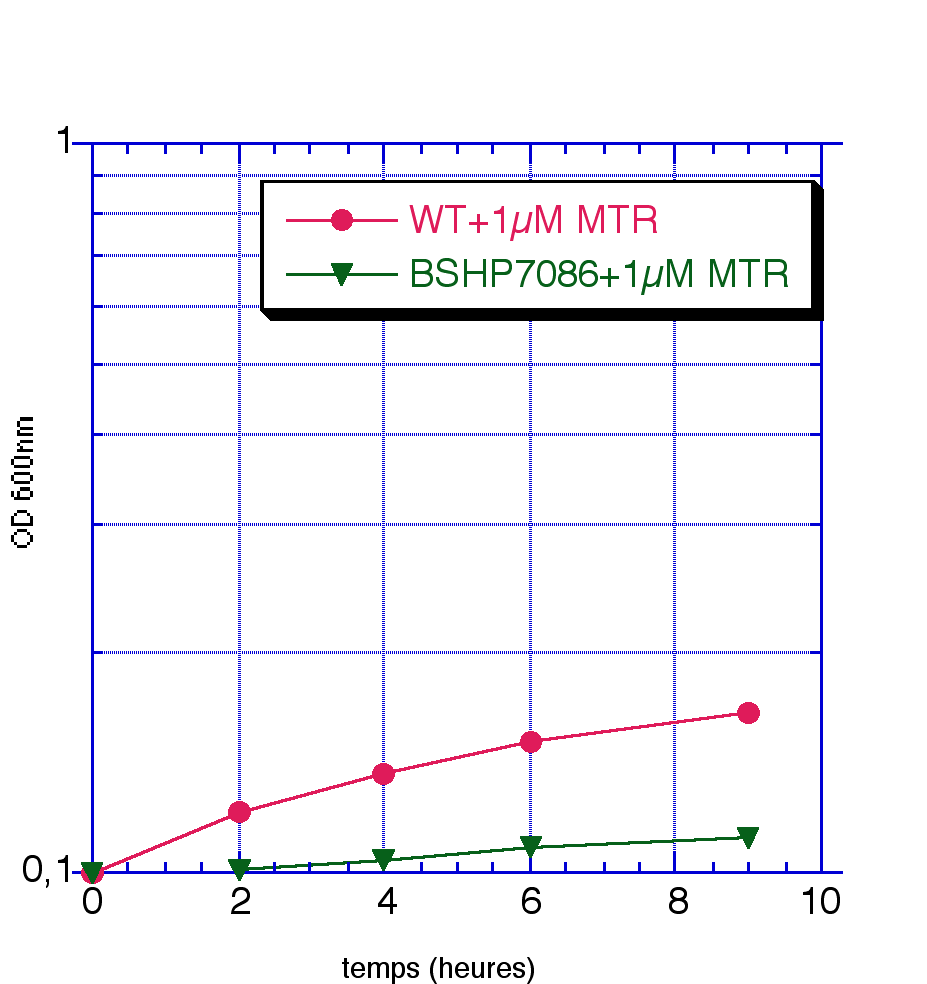


**Figure 2**. The growth of B.subtilis 168 WT and BSHP7086 (yfnA ΔrbsDACB nupQ) strains on 1 µM MTR as sole sulphur source.

Permease YhdG is a paralog of MtrA. The experiments reported in Figure 2 demonstrating that MTR is essentially transported by the action of three transporters, MtrA, the ribose transporter and NupQ, indicate that YhdG is unlikely to allow significant influx of MTR. In Lactococcus lactis its homolog has been shown biochemically to transport BCAA, while methionine inhibited BCAA transport (den Hengst et al., 2006). In B. subtilis, this permease has also been proposed to be a branched-chain aminoacids (BCAA) transporter, with preference for isoleucine and valine, and renamed BcaP accordingly (Belitsky, 2015). BcaP appears to be necessary to allow isoleucine and valine to be used as nitrogen sources. Because of its similarity to MtrA we nevertheless tested its possible role in MTR transport.

In sharp contrast with the situation with MtrA, in a medium devoid of BCAAs a deletion of bcaP resulted in a considerable enhancement of 3FMTR toxicity, showing that, at least functionally, this permease has a role linked to MTR metabolism. Our observation is compatible with a role as an antiporter that allows efflux of MTR or a derivative, likely to be trifluoromethylmethionine. Indeed, antiporters such as the lysine:cadaverine antiporter or the B. subtilis serine:threonine antiporter SteT display signifcant similarity with MtrA and BcaP. This hypothesis is substantiated by the fact that, exploring co-evolution with the PhyloProfile software (Engelen et al., 2012), we found that BcaP co-evolved with MtnB, a key step in the MSP, as well as with MtnU. The latter codes for the 2-ketoglutaramate omega amidase that prevents degradation of methionine, forcing its precursor 2-keto-3-methylthiobutyrate to be converted into methionine by the glutamine-dependent aminotransferase MtnE (Belda et al., 2013). We therefore surmise that BcaP is a BCAA:methionine antiporter. Further biochemical work will be needed to substantiate these observations.

**Identification of a key step in B. subtilis lysine biosynthesis**

Even within Proteobacteria, lysine biosynthesis is far from stemming from an ubiquitous pathway. Starting from a common precursor, tetrahydropicolinate, it proceeds via protection / deprotection steps of intermediary isomers of diaminopimelate (DAP) that are either succinylated or acetylated [for a picture of the likely phylogenetic complexity of similar steps, see methionine biosynthesis (Bastard et al., 2017)]. In Firmicutes, the succinylation step present in E. coli is replaced by an acetylation step, exactly as in methionine synthesis. A transamination reaction is required to generate N-acetyl-L,L-diaminopimelate which will be deacetylated (deprotection step) to L,L-diaminopimelate, subsequently delivering meso-diaminopimelate in the last step. In contrast to the enzymes described for the succinylase route, a number of enzymes of the acetylase branch have neither been purified nor characterized experimentally. Most of the corresponding knowledge was established in silico via comparative genomics and indirectly in vivo via genetic experiments. In B. subtilis the counterparts of the E. coli dapD, dapC and dapE genes were proposed to be genes ykuQ, patA and ykuR. While it was fairly straightforward to identify YkuQ as DapH (tetrahydrodipicolinate N-acetyltransferase) and YkuR as DapL (N-acetyl-L,L-diaminopimelate deacetylase), the activity of PatA (BSU14000) remained controversial. The only experimental knowledge about this genes showed that it was not a methionine transaminase (Berger et al., 2003), while deletion of the gene was first found to be dispensable (Feucht et al., 2003), then proposed to be essential (Tanaka et al., 2013).

We endeavoured to explore its function by inactivating the gene by antibiotic cassette replacement. We understood that this could however be lethal. Because we ignored whether B. subtilis could transport DAP, we grew the patA deletion strain on a hyperosmotic rich medium known to allow growth of cells lacking murein (Dominguez-Cuevas et al., 2012). This procedure allowed us obtain small colonies, of identical size whether in the presence or absence of DAP in the medium. In either case, when re-streaked on the same medium the cells kept growing. In contrast, when streaked on a medium with normal osmolarity, the cells failed to grow. This demonstrated both that the gene is essential for murein synthesis and that B. subtilis does not transport DAP, while possibly reconciling previous contradictory experiments (lethality or absence of lethality of the deletion). This latter observation was confirmed by checking the growth of B. subtilis on DAP as either a carbon or a nitrogen source. In eiçther cases we failed to observe any bacterial growth, indicating that B. subtilis missed the necessary DAP transport system. This situation is reminiscent of that observed in Corynebacterium glutamicum and Mycobacterium smegmatis, where DAP transport is missing or very slow (Pavelka and Jacobs, 1996).

Taking together the observations that the BSU14000 gene is essential for murein synthesis, that it must code for an aminotransferase, and that the only missing step in meso-DAP synthesis is N-acetyl-L,L-diaminopimelate aminotransferase, we can be confident that PatA is the missing enzyme. We therefore renamed patA as dapX.

**Experimental methods**

Bacterial strains and growth media

Escherichia coli and B. subtilis strains as well as plasmids used in this work are listed in Table 1. E. coli TG1 and XL1-Blue were used for cloning experiments (TG1 for single cross-over recombination and XL1-Blue for double cross-over recombination). E. coli and B. subtilis were grown in Luria-Bertani (LB) medium (Bertani, 1951) and in ED minimal medium (B. subtilis): K_2_HPO_4_, 8 mM; KH_2_PO_4_, 4.4 mM; glucose, 27 mM; Na_3_-citrate, 0.3 mM; L-glutamine, 15 mM; L-tryptophan, 0.244 mM; ferric citrate, 33.5 mM; MgSO_4_, 2 mM; MgCl_2_, 0.61 mM; CaCl_2_, 49.5 mM; FeCl_3_, 49.9 mM; MnCl_2_, 5.05 mM; ZnCl_2_, 12.4 mM; CuCl_2_, 2.52 mM; CoCl_2_, 2.5 mM; Na_2_MoO_4_, 2.48 mM. When MTR was used sole sulphur source, MgSO_4_ was replaced with MgCl_2_ at the same magnesium concentration (2 mM). When necessary the DAP (Sigma-Aldrich) was included at 1 mM concentration. LB and ED plates were prepared by addition of 17 g/liter Bacto agar or Agar Noble (Difco), respectively, to the medium. When included, antibiotics were added to the following concentrations: ampicillin, 100 mg/liter; chloramphenicol, 5 mg/liter; spectinomycin, 100 mg/liter; kanamycin, 10 mg/ml; erythromycin plus lincomycin, 1 mg/liter and 25 mg/liter. Bacteria were grown at 37°C. For the growth on hyperosmotic rich medium, the L-form medium was used (Dominguez-Cuevas et al., 2012). The optical density (OD) of bacterial cultures was measured at 600 nm.

##### Molecular genetics procedures

Plasmid DNA was prepared from E. coli by standard procedures (Sambrook and Russel, 2001). Bacillus subtilis chromosomal DNA was purified as described by Saunders (Saunders et al., 1984). Restriction enzymes and T4 DNA ligase were used as specified by manufacturers. DNA fragments used for cloning experiments were prepared by PCR using PfuUltra II Fusion HS DNA Polymerase (Agilent). Amplified fragments were purified by QIAquick PCR Purification Kit (Qiagen). DNA fragments were purified from a gel using Spin-X columns from Corning Costar by subsequent centrifugation and precipitation. Standard procedures were used to transform E. coli (Sambrook and Russel, 2001) and transformants were selected on LB plates containing ampicillin, spectinomycin or ampicillin plus spectinomycin. Bacillus subtilis cells were transformed with either plasmid DNA or linear PCR fragment DNA following the two-step protocol described previously (Kunst and Rapoport, 1995). Transformants were selected on either LB or L-form medium plates containing appropriate antibiotic. The proper insertion and/or deletion was verified by PCR using the RedTaq polymerase (Sima-Aldrich).

##### Mutants construction

The DNA downstream from the patA gene (nucleotides +123 to +427 relative to the translation start point) was amplified by PCR using primers introducing an EcoRI cloning site at the 5' end and a BamHI cloning site at the 3' end of the fragment, then inserted into the EcoRI and BamHI sites of plasmid pJM783 producing plasmid pAMA7011. This plasmid in which the patA gene was disrupted as well as fused (transcriptional fusion) with the lacZ gene was introduced into the chromosome of 168 strain by a single cross-over event, giving strain AMBS7035 (a non-viable strain on standard media).

To construct the rbsDACB deletion strain, a SmaI restricted spectinomycin resistance cassette was used (spc gene) (Murphy, 1985). Two DNA fragments, one upstream from the rbsD gene (nucleotides -120 to +312 relative to the translational start point of rbsD) and the second one downstream from the rbsB gene (nucleotides -189 to +317 relative to the rbsB stop codon) were amplified by PCR using primers introducing, for the first one, a EcoRI cloning site at the 5' end and a SmaI cloning site at the 3' end of the fragment, and for the second one, a SmaI cloning site at the 5'end and an BamHI site at the 3' end of the fragment. PCR products and the spectinomycin cassette were ligated and inserted into the EcoRI and BamHI sites of pUC19 (Boehringer Mannheim) producing the plasmid pAMA7012. Prior to tranformation this plasmid was linearized at its unique ScaI site. Complete deletion of the gene was obtained by a double cross-over event, giving strain BSHP7077.

To obtain the disruptant yufQ strain compatible with BSHP7083, the BFS1433 strain (yufQ::lacZ disruptant, from the European Union and Japanese consortium) was transformed with the ScaI linearized pEC23 plasmid (which carries a kanamycin resistance gene, M. Simon and P. Stragier, unpublished) for replacement with the kanamycin resistance gene of the lacZ and Erm genes belonging to the pMutin plasmid (erythromycin resistant) integrated into the genome, by homologous recombination. The resulting clones were checked for their inability to grow on erythromycin and chloramphenicol. The resulting strain was named BSHP7036. The chromosomal DNA of BSHP7036 was prepared and used to transform the BSHP7083 strain, producing the triple yfnA, rbsDACB and yufQ disrupted strain BSHP7086.

**Table 1.** Bacterial strains and plasmids used in this study.

| Strain or plasmid | Genotype or description | Source or reference |
| --- | --- | --- |
| Strains |  |  |
| Escherichia coli |  |  |
| TG1 | K12 supE hsdD5 thi Δ(lac-proAB) F'[traD36 proA^+^ proB+ lacI^q^ lacZΔM15] | Laboratory collection |
| XL1-Blue | K12 supE44 hsdR17 recA1 endA1 gyrA46 thi relA1 lac- F'[proAB+ lacI^q^ lacZΔM15 Tn10(tet^R^)] | Laboratory collection |
| Bacillus subtilis |  |  |
| 168 | trpC2 | (Spizizen, 1958) |
| YFNAd**^a^** | trpC2 yfnA::lacZ **^a^** | Functional analysis project**^a^** |
| YHDGd**^a^** | trpC2 yhdG::lacZ **^a^** | Functional analysis project**^a^** |
| BFS1433**^a^** | trpC2 yufQ::lacZ **^a^** | Functional analysis project**^a^** |
| BSHP7036 | trpC2 yufQ::aphA3 | This work |
| BSHP7077 | trpC2 rbsDACB::spc | This work |
| BSHP7094 | trpC2 yfnA yufQ::aphA3 | This work |
| BSHP7083 | trpC2 yfnA rbsDACB::spc | This work |
| BSHP7086 | trpC2 yfnA rbsDACB::spc yufQ::aphA3 | This work |
| AMBS7035 | trpC2 patA::lacZ | This work |
| Plasmids |  |  |
| pJM783 | cloning vector, Cm^R^, Amp^R^ | (Perego and Hoch, 1988) |
| pUC19 | cloning vector, Amp^R^ | (Yanisch-Perron et al., 1985) |
| pEC23 | integrative plasmid, Kan^R^ | M. Simon and P. Stragier, unpublished |
| pAMA7011 | pJM patA::lacZ | This work |
| pAMA7012 | pUC19 rbsDACB::spc | This work |

**a.** This strain has been constructed in the frame of the EC project for the functional characterization of the genome of B. subtilis in Europe (Kobayashi et al., 2003).

**b.** aphA3 is a kanamycin resistance gene, spc is the spectinomycin resistance gene from Staphylococcus aureus.

**References**

Albers, E. (2009) Metabolic characteristics and importance of the universal methionine salvage pathway recycling methionine from 5'-methylthioadenosine. IUBMB Life **61**: 1132-1142.

Bastard, K., Perret, A., Mariage, A., Bessonnet, T., Pinet-Turpault, A., Petit, J.L. et al. (2017) Parallel evolution of non-homologous isofunctional enzymes in methionine biosynthesis. Nat Chem Biol **13**: 858-866.

Belda, E., Sekowska, A., Le Fevre, F., Morgat, A., Mornico, D., Ouzounis, C. et al. (2013) An updated metabolic view of the Bacillus subtilis 168 genome. Microbiology **159**: 757-770.

Belitsky, B.R. (2015) Role of branched-chain amino acid transport in Bacillus subtilis CodY activity. J Bacteriol **197**: 1330-1338.

Berger, B.J., English, S., Chan, G., and Knodel, M.H. (2003) Methionine regeneration and aminotransferases in Bacillus subtilis, Bacillus cereus, and Bacillus anthracis. J Bacteriol **185**: 2418-2431.

Bertani, G. (1951) Studies on lysogenesis. I. The mode of phage liberation by lysogenic Escherichia coli. J Bacteriol **62**: 293-300.

den Hengst, C.D., Groeneveld, M., Kuipers, O.P., and Kok, J. (2006) Identification and functional characterization of the Lactococcus lactis CodY-regulated branched-chain amino acid permease BcaP (CtrA). J Bacteriol **188**: 3280-3289.

Dominguez-Cuevas, P., Mercier, R., Leaver, M., Kawai, Y., and Errington, J. (2012) The rod to L-form transition of Bacillus subtilis is limited by a requirement for the protoplast to escape from the cell wall sacculus. Mol Microbiol **83**: 52-66.

Engelen, S., Vallenet, D., Medigue, C., and Danchin, A. (2012) Distinct co-evolution patterns of genes associated to bacterial DNA polymerase III DnaE and PolC. BMC Genomics **13**: 69.

Feucht, A., Evans, L., and Errington, J. (2003) Identification of sporulation genes by genome-wide analysis of the sigmaE regulon of Bacillus subtilis. Microbiology **149**: 3023-3034.

Gianotti, A.J., Tower, P.A., Sheley, J.H., Conte, P.A., Spiro, C., Ferro, A.J. et al. (1990) Selective killing of Klebsiella pneumoniae by 5- trifluoromethylthioribose. Chemotherapeutic exploitation of the enzyme 5-methylthioribose kinase. J Biol Chem **265**: 831-837.

Kobayashi, K., Ehrlich, S.D., Albertini, A., Amati, G., Andersen, K.K., Arnaud, M. et al. (2003) Essential Bacillus subtilis genes. Proc Natl Acad Sci U S A **100**: 4678-4683.

Kunst, F., and Rapoport, G. (1995) Salt stress is an environmental signal affecting degradative enzyme synthesis in Bacillus subtilis. J Bacteriol **177**: 2403-2407.

Murphy, E. (1985) Nucleotide sequence of a spectinomycin adenyltransferase AAD(9) determinant from Staphylococcus aureus and its relationship to AAD(3')(9). Mol Gen Genet **200**: 33-39.

Nakano, T., Saito, Y., Yokota, A., and Ashida, H. (2013) Plausible novel ribose metabolism catalyzed by enzymes of the methionine salvage pathway in Bacillus subtilis. Biosci Biotechnol Biochem **77**: 1104-1107.

Pavelka, M.S., Jr., and Jacobs, W.R., Jr. (1996) Biosynthesis of diaminopimelate, the precursor of lysine and a component of peptidoglycan, is an essential function of Mycobacterium smegmatis. J Bacteriol **178**: 6496-6507.

Perego, M., and Hoch, J.A. (1988) Sequence analysis and regulation of the hpr locus, a regulatory gene for protease production and sporulation in Bacillus subtilis. J Bacteriol **170**: 2560-2567.

Sambrook, J., and Russel, D.W. (2001) Molecular cloning: a laboratory manual: Cold Spring Harbor Laboratory Press.

Saunders, C.W., Schmidt, B.J., Mirot, M.S., Thompson, L.D., and Guyer, M.S. (1984) Use of chromosomal integration in the establishment and expression of blaZ, a Staphylococcus aureus beta-lactamase gene, in Bacillus subtilis. J Bacteriol **157**: 718-726.

Schroeder, H.R., Barnes, C.J., Bohinski, R.C., Mumma, R.O., and Mallette, M.F. (1972) Isolation and identification of 5-methylthioribose from Escherichia coli B. Biochim Biophys Acta **273**: 254-264.

Sekowska, A., and Danchin, A. (2002) The methionine salvage pathway in Bacillus subtilis. BMC Microbiol **2**: 8.

Sekowska, A., Mulard, L., Krogh, S., Tse, J.K., and Danchin, A. (2001) MtnK, methylthioribose kinase, is a starvation-induced protein in Bacillus subtilis. BMC Microbiol **1**: 15.

Sekowska, A., Denervaud, V., Ashida, H., Michoud, K., Haas, D., Yokota, A., and Danchin, A. (2004) Bacterial variations on the methionine salvage pathway. BMC Microbiol **4**: 9.

Spizizen, J. (1958) Transformation of biochemically deficient strains of Bacillus subtilis by deoxyribonucleate. Proc Natl Acad Sci U S A **44**: 1072-1078.

Sun, J., Daniel, R., Wagner-Dobler, I., and Zeng, A.P. (2004) Is autoinducer-2 a universal signal for interspecies communication: a comparative genomic and phylogenetic analysis of the synthesis and signal transduction pathways. BMC Evol Biol **4**: 36.

Tanaka, K., Henry, C.S., Zinner, J.F., Jolivet, E., Cohoon, M.P., Xia, F. et al. (2013) Building the repertoire of dispensable chromosome regions in Bacillus subtilis entails major refinement of cognate large-scale metabolic model. Nucleic Acids Res **41**: 687-699.

Yanisch-Perron, C., Vieira, J., and Messing, J. (1985) Improved M13 phage cloning vectors and host strains: nucleotide sequences of the M13mp18 and pUC19 vectors. Gene **33**: 103-119.
